# *Anisakis simplex* products impair intestinal epithelial barrier function and occludin and zonula occludens-1 localisation in differentiated Caco-2 cells

**DOI:** 10.1371/journal.pntd.0008462

**Published:** 2020-07-06

**Authors:** Noelia Carballeda-Sangiao, Isabel Sánchez-Alonso, Alfonso Navas, Susana C. Arcos, Pilar Fernández de Palencia, Mercedes Careche, Miguel González-Muñoz

**Affiliations:** 1 Unit of Immunology, University Hospital La Paz Institute for Health Research (IdiPaz), Madrid, Spain; 2 Department of Products, Institute of Food Science, Technology and Nutrition (ICTAN), Agencia Estatal Consejo Superior de Investigaciones Científicas (CSIC), Madrid, Spain; 3 Department of Biodiversity and Evolutionary Biology, Museo Nacional de Ciencias Naturales (MNCN), CSIC, Madrid, Spain; Federal University of Minas Gerais, BRAZIL

## Abstract

**Background:**

*Anisakis* spp. are nematode parasites found in a wide range of marine organisms. Human beings may accidentally become infected, showing the symptoms of anisakiasis and allergic responses. There has been evidence of increased intestinal permeability in *A*. *simplex*–sensitized subjects and that specific IgE titres increase in some allergic patients when fishery products are re-introduced into their diet. The aims of this work were to study the effect of *A*. *simplex* crude extract on the intestinal integrity and permeability by using Caco-2 cell monolayer. To analyse the capacity of Ani s 4 allergen to cross the epithelial barrier.

**Methodology/Principal findings:**

Cellular bioenergetics, transepithelial electrical resistance, viability, permeability, reactive oxygen species generation and immunofluorescent staining of tight junction proteins were analysed. *A*. *simplex* crude extract compromises the Caco-2 cell monolayer integrity in a dose-dependent manner. This effect is detected at 1 hour of culture and integrity is recovered after 24 hours of culture. The epithelial barrier disruption is accompanied by an increase in paracellular permeability and reactive oxygen species production and by a delocalization of occludin and zonula occludens-1. Finally, Ani s 4, a thermostable and resistant to digestion allergen with cystatin activity, is able to cross the epithelial barrier in Caco-2 monolayer and reach a cumulative mean percentage of 22.7% of total concentration in the basolateral side after 24 hours of culture.

**Conclusions/Significance:**

Our results demonstrate that *A*. *simplex* induces an early and reversible alteration of integrity and permeability of Caco-2 cell monolayer and that an underlying mechanism of this effect would involve the oxidative stress and disruption of epithelial tight junctions. Additionally, it has been shown that Ani s 4 allergen is able to cross the epithelial barrier. These findings could explain the increased intestinal permeability observed in *Anisakis*-sensitized patients, the changes over time in IgE sensitization to *A*. *simplex* allergens, and the specific IgE persistence in *Anisakis* allergy.

## Author summary

The nematode *Anisakis simplex* is a parasite of marine mammals that can parasitize humans when a raw or undercooked fish containing live *A*. *simplex* larvae is consumed. As a result, gastrointestinal and/or allergic symptoms are reported. There has been evidence of increased intestinal permeability in *A*. *simplex*-sensitized subjects and serum levels of the specific immunoglobulin involved in allergic reactions (IgE) in some allergic patients when fishery products are re-introduced into their diet. These results suggest that *A*. *simplex* material present in fishery products could alter the normal gut function. We have analysed the effects of a cellular model of epithelial barrier exposed to *A*. *simplex* products. We have found that *A*. *simplex* induces an early and reversible alteration of integrity and permeability of epithelial cell monolayer and that an underlying mechanism of this effect would involve the oxidative stress and disruption of the proteins maintaining the barrier function. Additionally, it has been shown that Ani s 4, an *A*. *simplex* allergen, is able to cross the epithelial barrier. These findings could explain the increased intestinal permeability observed in *Anisakis*-sensitized patients, and the changes in specific IgE to *A*. *simplex* allergens when fishery products are re-introduced into their diet.

## Introduction

*Anisakis* spp. are nematode parasites found in a wide range of marine organisms. Their life cycle involves cetaceans and pinnipeds as final hosts, zooplankton as intermediate hosts, and fish and cephalopods as intermediate or paratenic hosts [1]. Species included in *Anisakis* genus are found worldwide [2–4]. Within the *Anisakis* spp. life cycle, humans may become accidental hosts in which the parasite can survive for a short period of time but cannot reproduce. Humans become infected by eating raw or undercooked fish that contain viable *Anisakis* spp. third-stage larvae. After being ingested, the *Anisakis* spp. larvae penetrate the gastric and intestinal mucosa, thus causing the symptoms of anisakiasis [5]. The severity of anisakiasis varies from mild to severe and can have gastric, intestinal, and ectopic forms. These parasites can also cause allergic responses. The principal allergic responses are gastro-allergic anisakiasis in which allergic symptoms are induced by parasite allergens in an acute gastric parasitism. Some authors claim that allergic reactions can also be elicited by the contamination of fishery products containing allergens with no necessity for live parasites to elicit the reactions [1].

The gut is the major organ at the interface of the internal and external environments and plays an important role in nutrient uptake, metabolism, and health, and a relationship between an altered intestinal permeability and the worsening of the clinical manifestations in patients with adverse reactions to food has been demonstrated [6–8]. Association of *Anisakis*-sensitization and of intestinal homeostasis has also been investigated. Increased intestinal permeability was evidenced in *A*. *simplex*–sensitized subjects with severe clinical symptoms that improved after six months of consuming a raw-fish-free diet [9]. These authors claimed that eating raw fish represents a high risk for the integrity of the intestinal mucosa. Furthermore, it has been reported that following sensitization to *Anisakis* spp. allergens, specific IgE titres increase in some allergic patients when fishery products are re-introduced into their diet [10].

On the other hand, *Anisakis* spp. have been involved in the modulation of cellular responses that could lead to a pathological condition. It has been reported that *A*. *pegreffii* crude extract (CE) is able to induce changes in the production of reactive oxidative species thus leading to inflammation and to modulate apoptosis related markers in fibroblast cell lines [11]. Additionally, when Caco-2 cells—an in-vitro model of the intestinal epithelial barrier—are exposed to *Anisakis* spp. CE, a marked expression of COX-2 is observed, showing that substances present in *Anisakis spp*. larvae can induce an inflammatory response in the intestinal epithelium [12].

Altogether, these results point to *Anisakis* spp. material present in fishery products could alter the normal gut function.

The aim of our work was to study the effect of *A*. *simplex* CE on intestinal integrity and permeability by using Caco-2 cell monolayer. Cellular bioenergetics, transepithelial electrical resistance, viability, permeability, ROS generation, and immunofluorescent staining of tight junction (TJ) proteins were analysed. The capacity of Ani s 4 allergen to cross the epithelial barrier was also analysed.

## Methods

### Preparation of *Anisakis simplex* crude extract

Live *A*. *simplex* larvae in the third stage (L3) were obtained from heavily infected fish ovaries and viscera at the central fish market in Madrid (Mercamadrid), Spain. Those fish were caught in the North East Atlantic fishing grounds. L3 were extracted from fish tissue, washed in PBS, and immediately frozen at –20°C until their use. Taxonomic identification was performed as previously described [13]. Then, L3 were ground in a Potter-ELV homogeniser and sonicated at 18w for 5s. CE was obtained after centrifugation of 16,000g at 4°C for 10min, sterile filtered, and lyophilised until their use. The protein concentration was determined by using Quick Start Bradford Protein Assay (Bio-Rad, Hercules, CA, USA) and using BSA as the standard.

Quantification of endotoxin in CE was performed with the Pierce Chromogenic Endotoxin Quant Kit (Thermo Fisher Scientific, Madrid, Spain) that uses amebocyte lysates derived from the blood of the horseshoe crab. The assay was carried out according to the manufacturer’s instructions.

### Caco-2 cell culture

The Caco-2 human colon-cancer cell line was obtained from the ECACC (European Collection of Authenticated Cell Cultures, Salisbury, UK). These cells were routinely grown in 75cm^2^ culture flasks (Corning, Madrid, Spain) in glucose-free Dulbecco’s Modified Eagle’s medium (DMEM, Gibco, Madrid, Spain) supplemented with 25mM galactose (Sigma-Aldrich, Madrid, Spain), 100U/mL penicillin, 100μg/mL streptomycin (Gibco), 1mM Na-pyruvate (Gibco), 2mM glutamax (Gibco), 25mM HEPES buffer (Gibco) and 10% heat-inactivated (56°C, 45min) fetal bovine serum (Gibco). The use of galactose instead of glucose as the energy substrate induces a cell’s energy phenotype relying more on mitochondrial ATP production than glycolysis and thus mimicking the *in vivo* oxidative intestinal phenotype [14]. Caco-2 cells were cultured at 37°C in a humidified atmosphere with 5% CO_2_ and up to 80–90% confluency and then were either subcultured or used for the experiments. Caco-2 cells with a passage number between 25 and 35 were used for the experiments.

### Mitochondrial respiration in Caco-2 Cells

Caco-2 cells (2.0x10^4^ cells/well) were cultured in Seahorse XF24 cell culture microplates (Agilent, Santa Clara, CA, USA) by using DMEM-galactose for 15 days. Differentiated cells were then incubated with *A*. *simplex* CE (0.5, 1 and 2mg/mL) for two hours. Thereafter, oxygen consumption rate (OCR) and extracellular acidification rate (ECAR) were measured with the XFe24 Extracellular Flux Analyser (Agilent) from the Cell Culture Service of Centro de Investigaciones Biológicas (CIB-CSIC). Basal OCR and ECAR were determined first. Then leak OCR and maximal ECAR were measured with 2μM oligomycin, maximal OCR with 2μM and 3μM carbonyl cyanide-p-trifluoromethoxyphenylhydrazone (FCCP, Sigma-Aldrich) and non-mitochondrial OCR with 2.5μM antimycin A and 1.25μM rotenone. The basal, leak, and maximal OCR values were corrected for non-mitochondrial OCR. OCR and ECAR values were normalized by cellular protein content as was analysed with the Pierce Rapid Gold BCA Protein Assay (Thermo Fisher Scientific). Four experiments in five replicates were performed.

### Transepithelial electrical resistance experiments

Caco-2 cells were seeded at a density of 1 × 10^5^ /cm^2^ cells in 12-well Transwell inserts of polycarbonate membrane with 0.4μm pore size (Corning). The cells were cultured by using DMEM-galactose for 15 days to reach differentiation, and the growth media were refreshed every three days. Transepithelial electrical resistance (TEER) was monitored by using a MilliCell-ERS-2 volt-ohm-meter (Merck Millipore, Madrid, Spain). Only the monolayers with TEER > 1,000Ω·cm^−2^ were used for Caco-2 monolayer permeability experiments.

To analyse the effect of CE on TEER, Caco-2 cells cultured for 15 days were exposed to 2, 1, and 0.5mg/mL CE, and TEER was measured at different time points. To determine whether changes on TEER were influenced by thermostable components of CE, the extract was boiled for 30 minutes, and the effect of 2mg/mL CE on TEER was recorded. Other similar experiments were performed with 2 and 1mg/mL CE in the presence of 30μg/mL polymyxin B (Sigma-Aldrich), an antibiotic that binds and neutralizes lipopolysaccharides [15–18], to assess the putative effect on TEER of trace amount of endotoxin present in CE. To analyse if the effect of CE on TEER was dependent on proteases, cells were cultured with 2mg/mL CE in the presence of Protease Inhibitor Cocktail (Sigma-Aldrich, P2714). A 10X stock solution was prepared according to the manufacturer’s recommendations and then diluted 1:10 in the CE. TEER was recorded at 1, 2, 4, 6, and 24 hours in all experiments. Four experiments in triplicates were performed.

### Cell viability

Caco-2 cells were seeded at 5x10^4^ /well in a 96 well plate (Corning) and incubated for 24 hours at 37°C, 5% CO_2_. Thereafter, the cells were exposed to 2 and 0.5mg/mL CE for two and four hours and viability was analysed by using the Cell Counting Kit-8 (CCK-8, Sigma-Aldrich), which uses the highly water-soluble tetrazolium salt WST-8 [2-(2-methoxy-4-nitrophenyl)-3-(4-nitrophenyl)-5-(2,4-disulfophenyl)-2H-tetrazolium monosodium salt]. The assay was conducted according the manufacturer’s instructions. Experiments in triplicates were performed.

### Caco-2 cell monolayer permeability

Caco-2 cells were cultured for 15 days in 12-well Transwell inserts as described above. For measurement of the effect of 2 and 1mg/mL CE on the apical-to-basolateral permeability, 200μg/mL Lucifer Yellow (LY, Sigma-Aldrich) was added to the apical compartment. After two and four hours of incubation, samples from the basolateral compartment were analysed for the presence of LY with a fluorescence spectrophotometer at 410/540nm. The results are expressed as the percentage of the starting amount that permeated the monolayer. Three experiments in triplicates were performed.

The transport of the allergen Ani s 4 was assessed by western blot. For this purpose, Caco-2 cells were cultured with 2mg/mL CE in Transwell inserts, and a whole medium was collected and refreshed from the basolateral side at different time points. The samples were subjected to SDS-PAGE at 120V on 4%–20% Tris-glycine gel (Bio-Rad). Thereafter, they were transferred to nitrocellulose membranes by applying a constant current of 1.3A for 7min in a Trans-Blot Turbo Instrument (Bio-Rad). Membranes were blocked with PBS, 0.05% Tween 20, and 1% BSA for 1h at room temperature and then incubated with a rabbit anti-Ani s 4 polyclonal antiserum (1/10,000 dilution, 1 hour, room temperature) and subsequently with alkaline-phosphatase conjugated goat anti-rabbit IgG (1/5,000 dilution, 30 minutes, room temperature; BioSource International, Camarillo, CA, USA). The results are expressed as the percentage of the starting amount that permeated the monolayer. Four separate experiments were performed.

### Evaluation of intracellular ROS

Caco-2 cells were incubated at 5x10^4^ cells/well on 96-well plates and allowed to attach overnight. Then, they were stained with the cell-permeant reagent 2’, 7’-dichlorofluorescin diacetate (DCFDA) and incubated with 2, 1, and 0.5 mg/mL of *A*. *simplex* CE for one and four hours. Thereafter, the cells were lysed, and the fluorescence was read with a fluorometric plate reader at 480nm/530nm. Three experiments in triplicates were performed.

### Cell culture with N-acetylcysteine

Caco-2 cells were cultured for 15 days in 12-well Transwell inserts as described above. The cells were incubated with the antioxidant N-acetylcysteine (NAC; 4mM) for one hour. Thereafter, they were exposed to 2mg/mL *A*. *simplex* CE in the presence of 4mM NAC, and TEER was measured at 1, 4, and 24 hours. Three experiments in triplicates were performed.

### Immunofluorescent staining of tight junction proteins

After 15 days of differentiation, Caco-2 on glass coverslips were incubated with 2 and 1mg/mL *A*. *simplex* CE and fixed in 3% formaldehyde at two and 24 hours. Thereafter, the Caco-2 cells were permeabilized with 0.1% TX-100, 0.2% BSA in PBS for 10 minutes and then blocked with 2% BSA in PBS for one hour. Subsequently, the cells were incubated with rabbit anti-ZO-1 polyclonal antibody (40–2200, 1:100, Thermo Fisher Scientific) and mouse anti-occludin monoclonal antibody (OC-3F10, 1:200, Thermo Fisher Scientific) for 90 minutes. Then, the cells were incubated with Alexa Fluor-488-labelled goat-anti-rabbit IgG (H+L) (A-11008, 1:1,000, Thermo Fisher Scientific) and Alexa Fluor-647-labelled goat anti-mouse IgG (H+L) (A-21235, 1:500, Thermo Fisher Scientific) as secondary antibodies for one hour. Localization and distribution of TJ proteins were analysed with a Leica TCS SP5 confocal microscope (Mannheim, Germany) with an AOBS (Acousto-Optical Beam Splitter) with 40X and 63X oil immersion optics. Laser lines at 488nm and 633nm were provided by an Argon laser and a HeNe laser. Detection ranges were set to eliminate crosstalk between fluorophores. Four experiments were performed. Assays were performed in the Confocal Laser and Multidimensional Microscopy in vivo Service of CIB-CSIC.

### Statistical analysis

Data are expressed as mean ± standard deviation. Mean comparisons were performed with the t Student test. Differences in OCR values were analysed with the Kruskal-Wallis H test. Differences were considered significant when the *P*-value was <0.05. Statistical analyses were performed using SPSS 20.0 software (IBM Corporation, NY, USA).

### UNE-EN ISO 9001 certification

The Institute of Food Science, Technology and Nutrition (ICTAN-CSIC) has been certified under UNE-EN ISO 9001 for “Management and execution of research projects and contracts in the area of Food Science and Technology and Nutrition” (certificate number ER-0366/2015).

### Ethics statement

Ethics approval is not applicable since hakes used in this study were caught for commercial aims, more specifically for human consumption, and not with research aims so that the capture of the animals was not part of the experimental activity proposed in this work. The experimental design did not involve the manipulation of the live animals but of tissues (i.e. viscera parasitized with *Anisakis* L3) extracted from already non-living specimens acquired in a local fish monger and in a Central Fish Market

## Results

### *Anisakis simplex* CE impaired Caco-2 monolayer integrity

**Fig 1:**
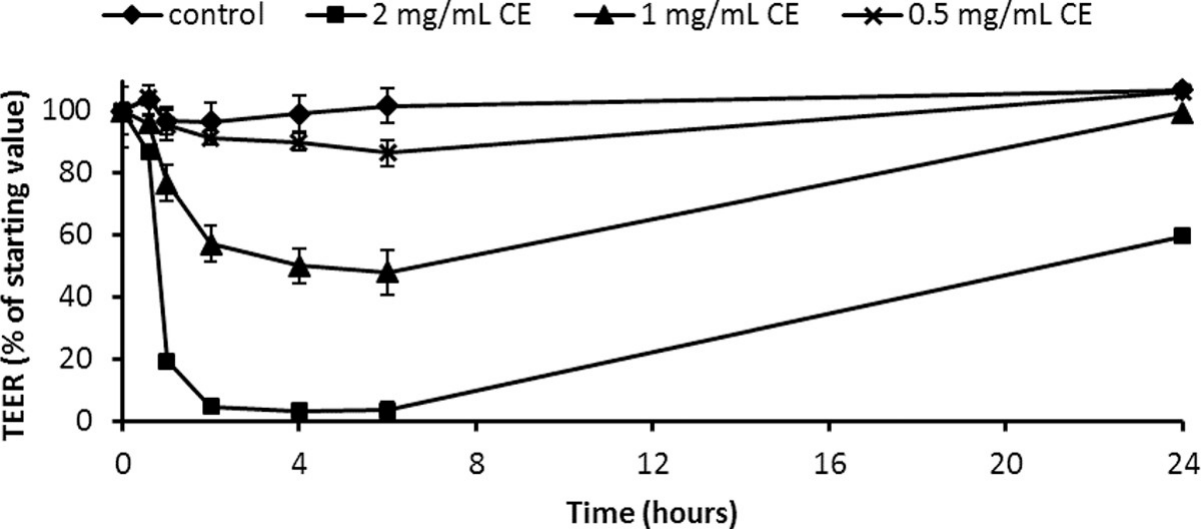
Changes in transepithelial electrical resistance induced by *A*. *simplex* crude extract. Caco-2 cells were cultured using DMEM-galactose for 15 days to reach differentiation and were exposed to 2, 1 and 0.5 mg/mL CE. TEER was measured at different time points and the results are expressed as mean ± SD.

**Fig 2:**
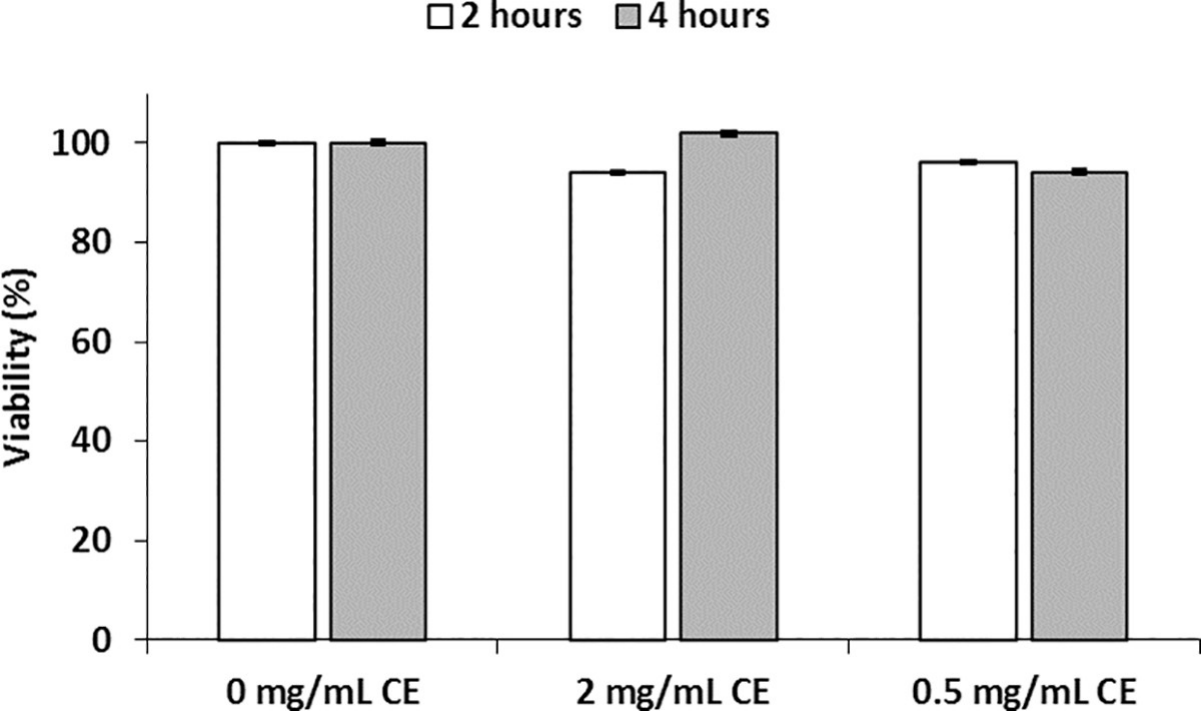
Effect of *A*. *simplex* crud extract on viability of Caco-2 cells. Caco-2 cells were seeded in a 96 well plate and incubated for 24 hours at 37°C, 5% CO2. Thereafter, cells were exposed to 2 and 0.5mg/mL CE for 2 and 4 hours and viability was analysed with the water-soluble tetrazolium salt WST-8. The results are expressed as mean ± SD. There were not significant differences between 0 mg/mL and 2 mg/mL 2 hours (P = 0.397) and 4 hours (P = 0.333), and between 0 mg/mL and 0.5 mg/mL 2 hours (P = 0.624) and 4 hours (P = 0.738).

To assess whether *A*. *simplex* proteins modify Caco-2 monolayer integrity, TEER was measured in cells differentiated in DMEM-galactose and cultured with parasite CE. *A*. *simplex* CE decreased TEER in a dose-dependent manner that was more remarkable with 2mg/mL CE. This effect was observable first at one hour and was maximal at 4–6 hours of exposure to CE. TEER recovered completely after 24 hours of culture with 0.5mg/mL CE while recovery reached about 90% and 50% of the starting value with 1 and 2mg/mL CE, respectively (Fig 1). To evaluate whether the CE-induced TEER decrease was related to a decrease in cellular viability, Caco-2 cells viability was analysed in cultures with the two CE concentrations causing maximal and minimal effects in TEER (2 and 0.5mg/mL) for two and four hours. The results indicate that the decrease in TEER could not be explained by cytotoxicity (Fig 2).

Quantification of endotoxin in the *A*. *simplex* CE shows that the 2mg/mL CE preparation contained trace amounts of lipopolysaccharides (LPS) (0.017ng/mL). To evaluate whether this endotoxin level would account for the effect in TEER, the Caco-2 cells were cultured with 2 and 1mg/mL CE in the presence of 30μg/mL polymyxin B to neutralize the endotoxin. The results showed that the profile of changes in TEER induced by the CE was not related to the LPS trace (S1 Fig).

Experiments were performed to explore the mechanisms underlying these changes in TEER. To determine whether parasite enzymes present in the CE could be involved in the TEER decrease by enzymatic degradation of TJ proteins, the cells were cultured with 2mg/mL of CE in the presence of protease inhibitors. No significant changes in the decrease in TEER were observed after one and four hours of culture as compared with the control culture (S2 Fig). Similar observations were recorded when CE was previously boiled to be incubated with the Caco-2 cells (S2 Fig). These results showed that the factors inducing the decrease in TEER were thermostable and independent of parasite protease activity.

### *Anisakis simplex* CE did not impair cellular respiration

**Fig 3:**
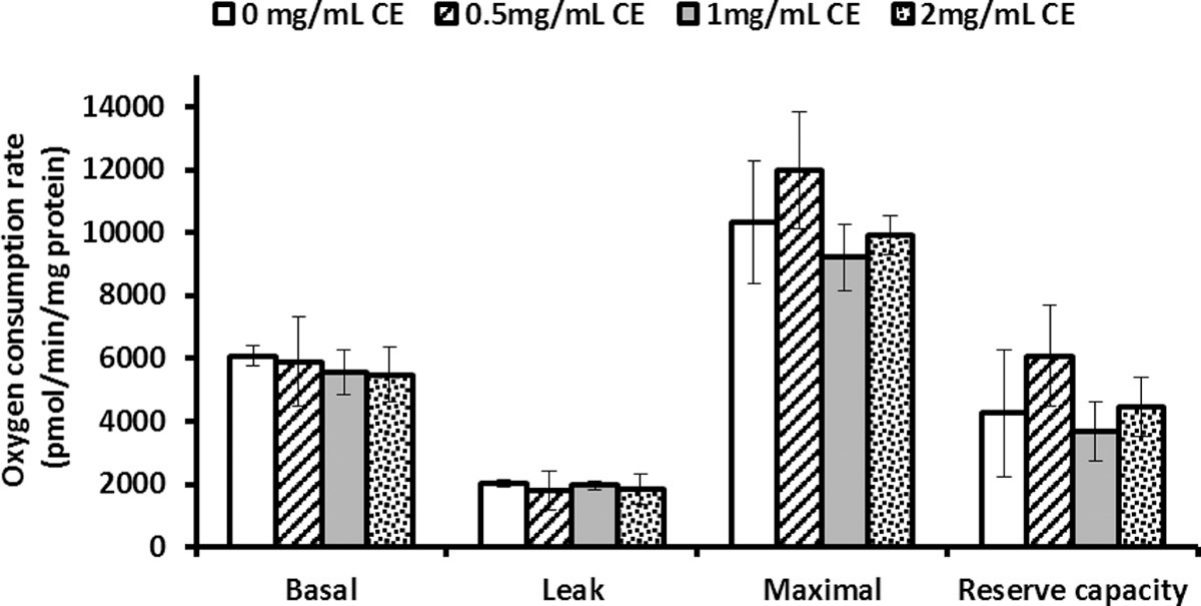
Basal, leak and maximal oxygen consumption rates and reserve capacity in Caco-2 monolayer exposed to *A*. *simplex* CE. Caco-2 cells were cultured using DMEM-galactose for 15 days. Differentiated cells were then incubated with A. simplex CE (0.5, 1 and 2 mg/mL) for 2 hours and basal, leak, maximal oxygen consumption rates and reserve capacity were determined. The results are expressed as mean ± SD. No significant differences were found in basal (P = 0.636), leak (P = 0.690) and maximal (P = 0.097) OCR and reserve capacity (P = 0.124).

To assess whether the TEER decrease was associated with changes in cellular respiration, different parameters were measured by live-cell metabolic assays in Caco-2 cells incubated with several concentrations of *A*. *simplex* CE. Rates of O_2_ consumption and glycolysis (ECAR) were determined by sequential addition of inhibitors to probe the function of individual components of the respiratory chain (S3 and S4 Figs). Basal rates were measured after adding 2, 1, or 0.5mg/mL of *A*. *simplex* CE to Caco-2 cells. To estimate the OCR coupled to ATP synthesis, oligomycin was next injected, and as expected, the OCR rate decreased in all the samples where the OCR consumption was due to proton leak of about 30% of basal OCR. The maximal OCR was then determined by injecting FCCP, and both controls and *A*. *simplex* CE-exposed Caco-2 showed a similar increase in O_2_ consumption. Finally, non-mitochondrial OCR was determined by injecting actimycin A and rotenone (S3 and S4 Figs). Profiles of OCR expressed as pmol 0_2_/min/mg protein showed no statistical differences in basal OCR, proton leak, maximal OCR, or reserve capacity for bioenergetics function (maximal OCR minus basal OCR) between Caco-2 exposed to *A*. *simplex* CE and controls (Fig 3).

### Caco-2 monolayer permeability

**Fig 4:**
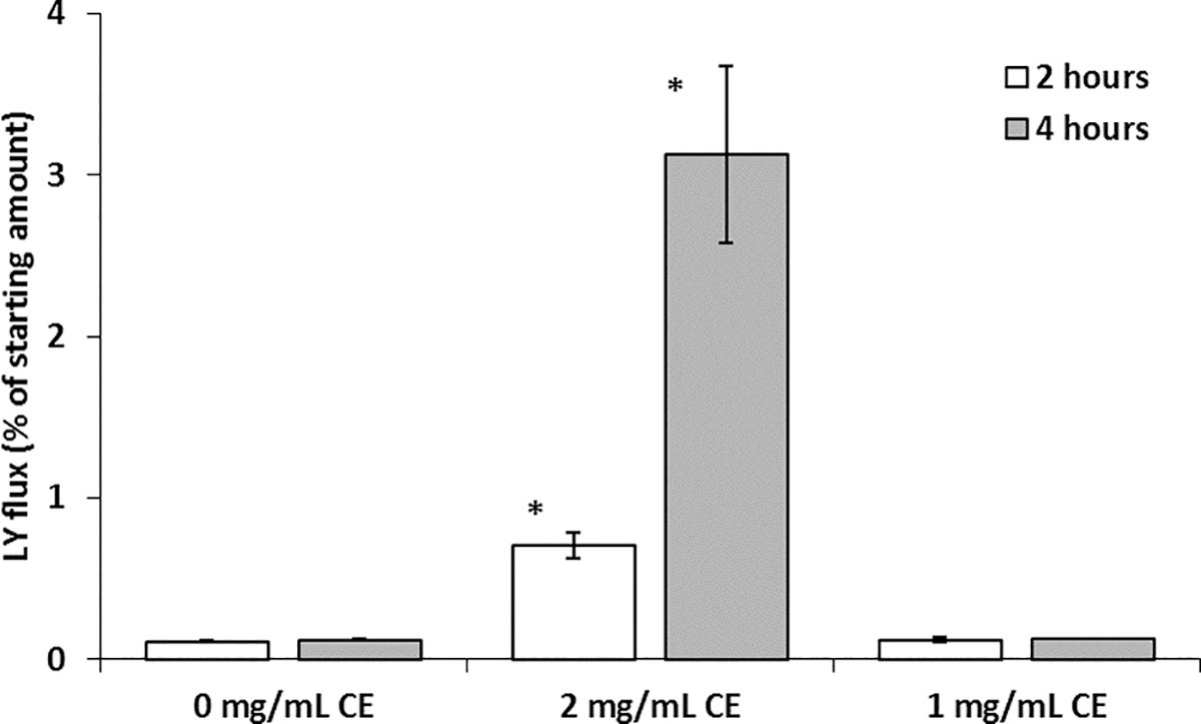
Effect of *A*. *simplex* CE on the paracellular permeability in Caco-2 cell monolayers. Caco-2 cell monolayers were exposed to 2 and 1 mg/mL CE. Lucifer yellow was added to the apical compartment and fluorescence in basolateral side was measured after 2 and 4 hours of incubation. The results are expressed as median ± SD and the asterisk indicates significant differences between 0mg/mL and 2 mg/mL for 2 hours (P<0.001, 95%CI = -0.65—-0.54) and 4 hours (P< 0.001, 95%CI = -3.6—-2.4).

To analyse whether the changes in TEER would lead to an increase in permeability, the flux of LY from apical to basolateral compartments in the Caco-2 cell monolayer cultured in the presence of CE was determined. Fluorescence was evaluated in Caco-2 cultured with 2 and 1mg/mL CE for two and four hours. A time-dependent flux of LY was found in the cultures with 2mg/mL indicating that the increase in the paracellular permeability allowed the passage of that marker molecule (Fig 4).

**Fig 5:**
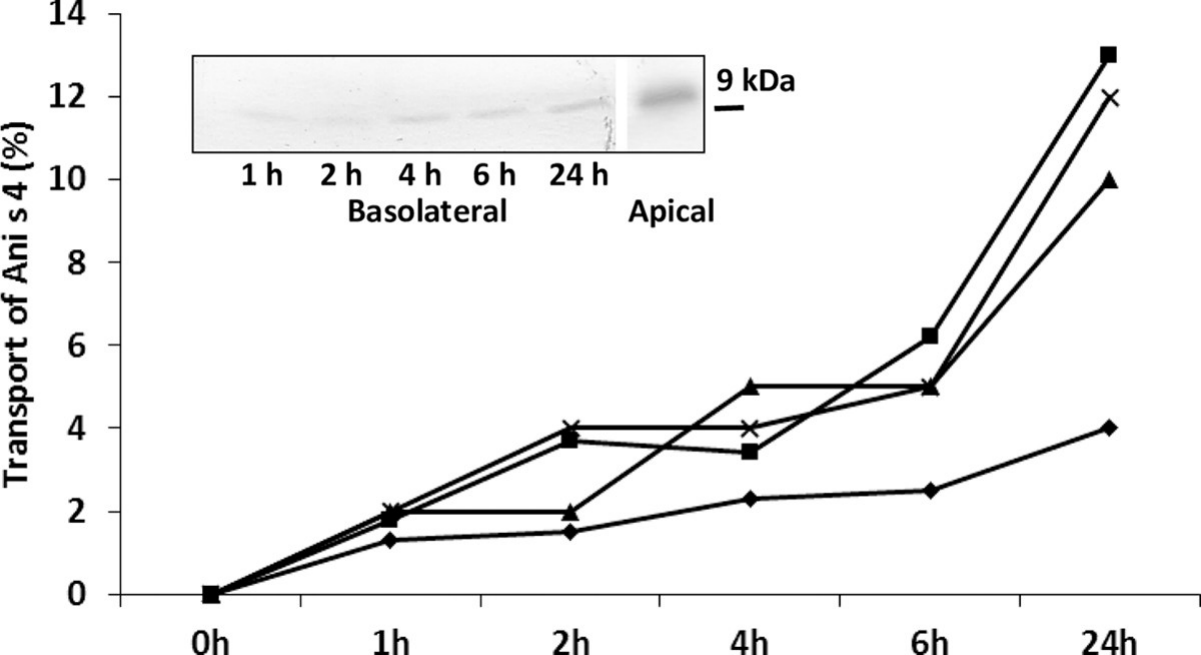
Kinetics of Ani s 4 transfer across Caco-2 monolayer. Caco-2 cells were incubated with 2mg/mL *A*. *simplex* CE. Basolateral and apical compartments were analysed for the presence of Ani s 4 at indicated time points. Results are expressed as percentage of total concentration (apical + basolateral compartments) of Ani s 4. Lines denote results from independent experiments. Western blot of a representative case is shown (experiment ▲).

On the other hand, the Caco-2 monolayer permeability to *A*. *simplex* allergen was analysed by using the Ani s 4 as a marker of allergen permeation. Ani s 4 was already detected in the basolateral compartment at one hour of culture, and the transport was maintained throughout the duration of the culture. Mean percentage values of the Ani s 4 detected in four independent experiments were 1.7, 2.8, 3.7, 4.7, and 9.5 at 1, 2, 4, 6, and 24 hours of culture respectively (Fig 5). The cumulative mean percentage of Ani s 4 transported was 22.7% of total concentration (apical + basolateral compartments).

### *Anisakis simplex* CE induced oxidative stress in Caco-2 cells

**Fig 6:**
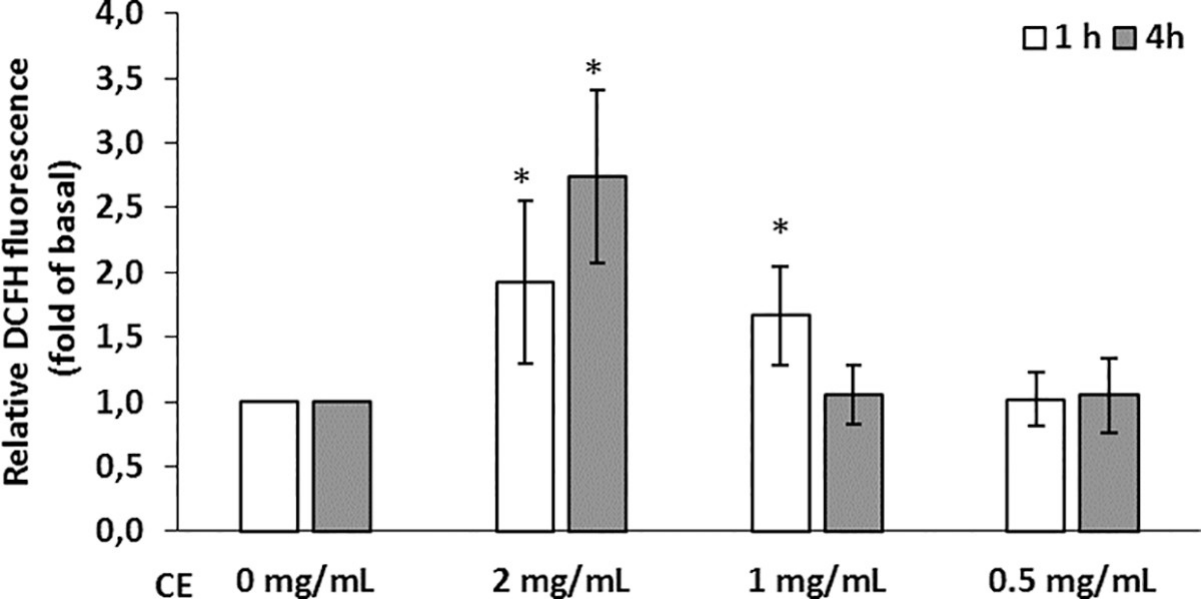
*A*. *simplex* crude extract leads to oxidative stress. Reactive oxygen species production, expressed as relative fluorescence, in Caco-2 cells after incubation for 1 and 4 hours with 2, 1 and 0.5mg/mL CE. The results are expressed as mean ± SD. The asterisks indicate significant differences between the control (0mg/mL) and 2mg/mL 1hour (P = 0.048, 95%CI = -1.8—-0.1) and 4 hours (P = 0.008, 95%CI = -2.9—-0.75), and 1mg/mL 1hour (P = 0.036, 95%CI = -1.2—-0.5).

**Fig 7:**
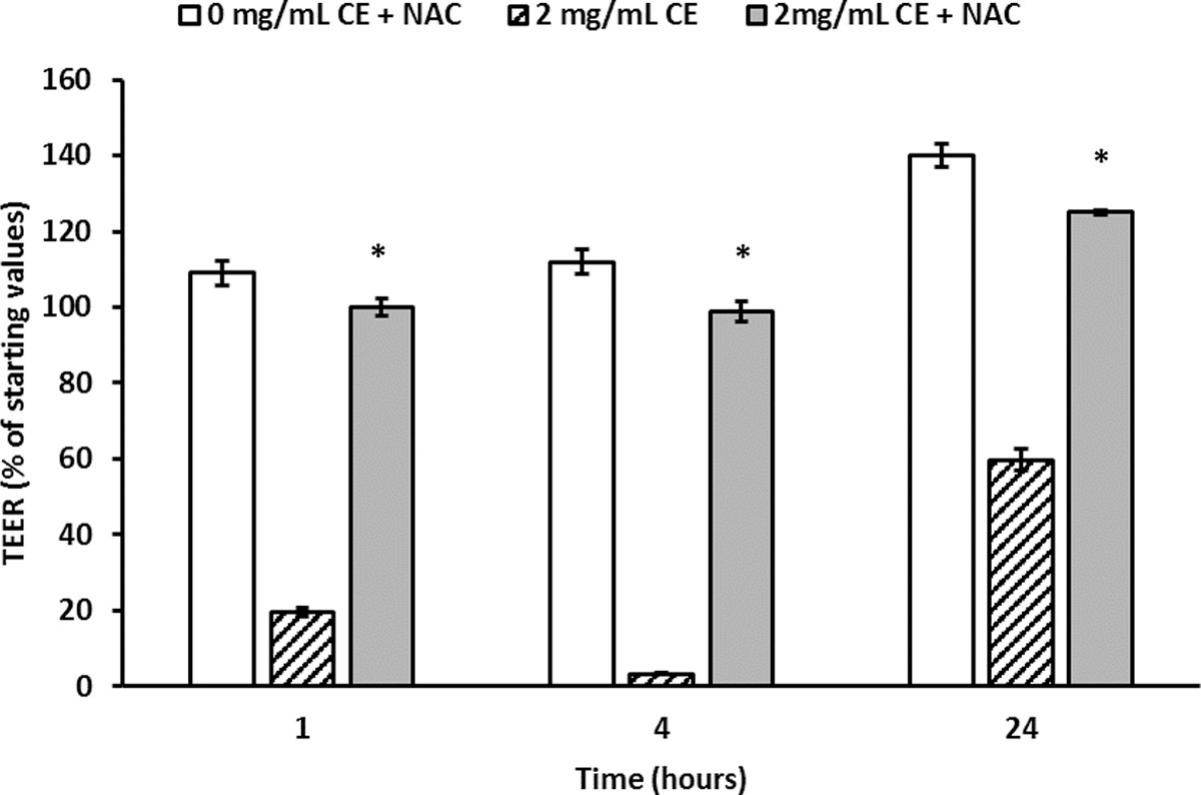
N-acetilcysteine prevents the *A*. *simplex* CE-induced TEER decrease. Caco-2 cell monolayers were incubated with the antioxidant N-acetylcysteine (NAC; 4mM) for 1 hour. Thereafter, cells were exposed to 2mg/mL *A*. *simplex* CE in the presence of 4mM NAC and TEER was measured at 1, 4 and 24 hours. The results are expressed as mean ± SD. The asterisks indicate significant differences between absence and presence of NAC 1hour (P<0.001, 95%CI = -74.6—-69.3), 4 hours (P<0.001, 95%CI = -88.0—-81.8) and 24 hours (P = 0.002. 95%CI = -30.8—-12.8).

ROS production was determined in Caco-2 cells exposed to 2, 1, and 0.5mg/mL of *A*. *simplex* CE. A significant increase in ROS production was observed in cells treated with 2 and 1mg/mL of CE compared with those of the untreated control. Conversely, the cells treated with 0.5mg/mL of CE show unchanged ROS production (Fig 6). To assess whether the antioxidant NAC could protect them from the increase in ROS production, cells were pre-treated with NAC and then exposed to 2mg/mL of CE in the presence of NAC. The results showed that NAC prevents the *A*. *simplex* CE-induced TEER decrease (Fig 7).

### *Anisakis simplex* CE disrupted intercellular tight junctions

**Fig 8:**
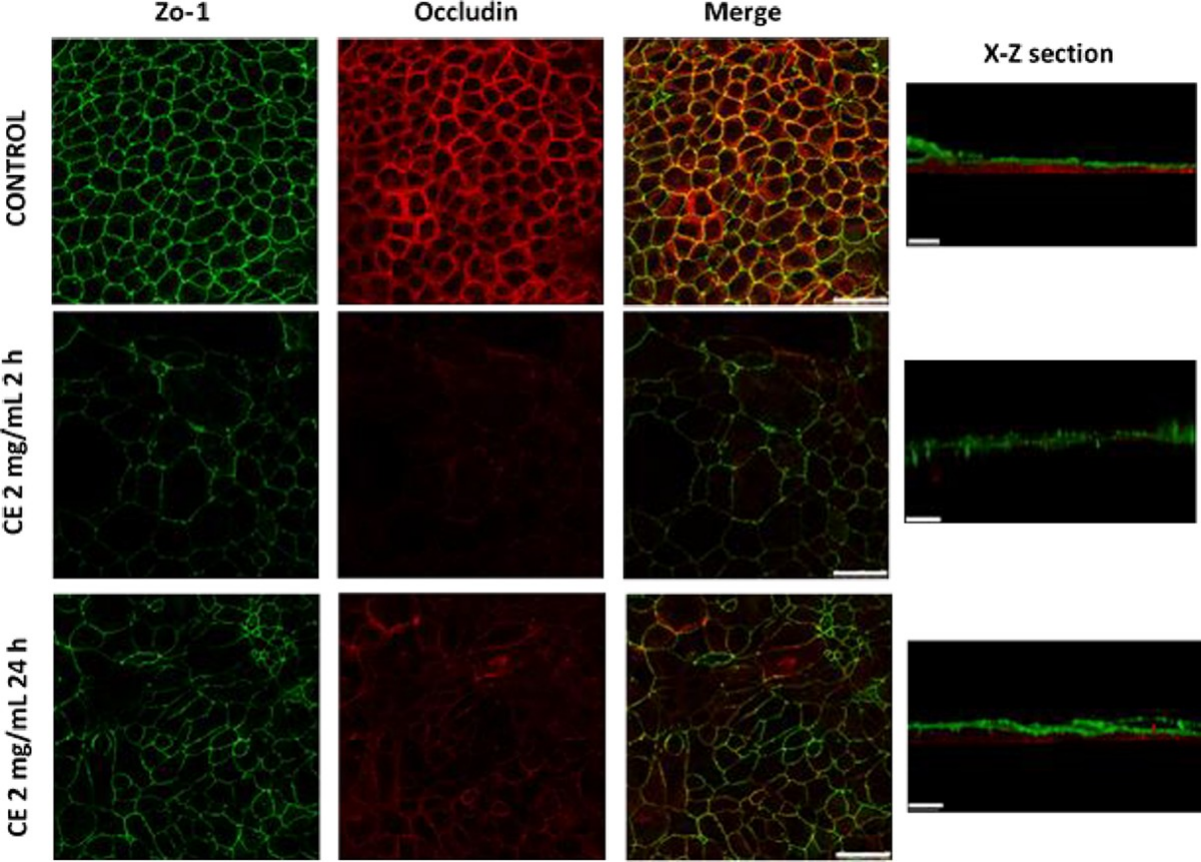
Effects of *A*. *simplex* CE on TJ proteins. Caco-2 cells were incubated with medium or 2mg/mL CE and, thereafter, immunofluorescence staining was performed to localize ZO-1 (green) and occludin (red). TJ proteins distribution is shown at the time of maximal decrease of TEER (2 hours) and recovery of TEER (24 hours). Scale bar represents 50 μm (x-y section) or 20 μm (x-z section).

The location of proteins maintaining TJ was analysed subsequent to the incubation of Caco-2 cells with 2mg/mL *A*. *simplex* CE at various time points with a maximum decrease of TEER (> 90% at 2 hours) and partial recovery of TEER (> 50% at 24 hours). In the control samples, ZO-1 and occludin showed typical cellular staining. At two hours of incubation with CE, TJ rings became broken. Changes were more extensive for occludin, which was barely detected. In x–z sections, the pattern of ZO-1 appears as a broken and thicker staining line while occludin is not observed. After 24 hours of incubation with CE, the normal distribution of TJ proteins was partially recovered (Fig 8).

## Discussion

This study has demonstrated that *A*. *simplex* products alter the integrity and barrier function of Caco-2 monolayer cultures and that these effects are associated with increased ROS production and changes in TJ protein localization.

The food-borne parasite *Anisakis* spp. is able to parasitize human beings accidentally when raw or undercooked parasitized fish is consumed. As a result, subjects show gastrointestinal symptoms, i.e., anisakiasis, and occasionally concurrent allergic symptoms, i.e., gastroallergic anisakiasis [5, 19]. Furthermore, there is evidence that parasite products in fish are involved in the occurrence of allergic symptoms with no necessity for live parasites [1, 10, 20, 21]. This implies that fish-borne parasite products would reach the intestinal tract and trigger the local immune system. It has previously been reported that the alimentary habit of eating raw fish represents a high risk for the integrity of the intestinal mucosa [9]. Furthermore, *Anisakis* spp.-specific IgE monitoring shows that specific IgE titres increase in some allergic patients when frozen fish is introduced into their diet [10]. On the other hand, experiments on the small intestine exposed to *A*. *simplex* CE or excretory/secretory products show that parasite products cause an autonomic imbalance in the gut [22].

It is thought that intestinal barrier dysfunction plays a role in both sensitisation and effector phases of food allergy. There is evidence of increased intestinal permeability in patients with food allergy as compared with that of control patients [23, 24]. Caco-2 monolayers have been extensively used as an experimental model to determine the mechanisms underlying the intestinal epithelial barrier dysfunction in food allergy [25]. The kiwifruit allergen Act d 1 reduces TEER in cell monolayers and increases intestinal permeability to β-lactoglobulin or dextran in animal models [26, 27]. The effect of Act d 1 on intestinal barrier integrity depends on its cysteine protease activity. The allergenic potential of proteases is related to their capacity to hydrolyse the proteins responsible for maintaining the epithelial intercellular junctions. This is also the case of the allergen Der p 1, a serine protease from house dust mite able to promote its transepithelial movement mediated by its own protease activity [28]. As the proteolytic activity of allergens themselves seems to confer enhanced sensitizing potential by disruption of epithelial barrier layers, and also given that *Anisakis* spp. contain a number of proteases that play a significant role in their life cycle and parasite-host relationship [29, 30], the putative involvement of the parasite protease activity in the decrease in TEER was investigated by enzymatic inhibition assays. Our results show that protease inhibition does not prevent integrity dysfunction in Caco-2 monolayer as determined by TEER.

The early decrease in TEER observed in Caco-2 monolayer could be explained by a putative effect of the CE on the cell viability. In fact, *A*. *pegreffii* CE causes a remarkable loss of viability in a fibroblast cell line in a time- and protein-concentration-dependent manner [11]. We have analysed Caco-2 cell viability incubated with 2 and 0.5mg/mL of CE at time points corresponding to the maximal TEER decrease and no deleterious effect was observed. Differences among these studies could be explained because the viability analysis was performed at different time points or because different cell lines have been used.

Another factor that has been involved in the maintenance of the intestinal barrier function is the mitochondrial ATP production. For instance, non-steroid anti-inflammatory drugs are thought to induce intestinal permeability by uncoupling oxidative phosphorylation, which leads to mitochondrial dysfunction and consequently to a decrease in intracellular ATP [31, 32]. On the other hand, it has been reported that inhibition of mitochondrial ATP production increases Caco-2 monolayer permeability following the use of an *in-vitro* model mimicking the oxidative *in vivo* situation [14]. To analyse whether *A*. *simplex* CE could alter the cellular bioenergetics and thus contribute to the integrity barrier dysfunction, the cellular respiration was measured since it is a tool to assess energy metabolism owing to the coupling between ATP synthesis and oxygen consumption during oxidative phosphorylation. Our results show no significant differences in basal respiration, ATP-linked respiration, proton-leak-linked respiration, or maximal respiration between Caco-2 cells cultured with and without CE. Accordingly, the reserve capacity (maximal OCR minus basal OCR) remains unchanged after incubation with CE, which suggests that Caco-2 cells can appropriately respond to an increased ATP demand and withstand periods of stress [33].

The decrease in TEER induced by *A*. *simplex* CE reflects the ionic conductance of the paracellular pathway in the Caco-2 monolayer [34]. We have used LY, a compound transported via the paracellular pathway, as a non-electrolyte tracer to analyse the apical-basolateral passage [35]. LY was detected in the basolateral compartment coincident with the maximal decrease in TEER of 2mg/mL CE, thus indicating an increase in the paracellular water flow [34].

The capacity of allergens to cross the intestinal barrier is thought to be related to their allergenic potential and allergy persistence [8, 36, 37]. We have analysed whether the Ani s 4 allergen was transported to the basolateral side. Ani s 4 is a thermostable protein resistant to pepsin digestion and represents a clinically relevant allergen in *A*. *simplex* allergy [38, 39]. Ani s 4 was detected in the basolateral compartment throughout the culture period and was detected as a 9kDa protein band by western blotting thus suggesting that the allergen is resistant to the enzymes located in the endocytic vesicles and lysosomes [40] of Caco-2 cells. Other allergens have also been demonstrated to translocate through the Caco-2 monolayer while maintaining their primary protein structure as Pru p 3, the peach lipid transfer protein [41], and the peanut allergens Ara h 1 and Ara h 2 [42]. One may think that Ani s 4 is not the only parasite allergen able to cross the intestinal barrier and that this ability could be related to the increase of *Anisakis spp*.-specific IgE in some sensitized individuals consuming previously frozen fishery products, that is, with no live larvae [10]. Assessment of the relative contribution of paracellular and transcellular pathways to the translocation of Ani s 4 requires further investigation.

Previous research efforts have shown that *A*. *pegreffii* modulate ROS generation in fibroblast cell lines [11] and *Anisakis* spp. induced a marked COX-2 expression in Caco-2 cells [12]. Our results show that *A*. *simplex* CE induces an early increase in ROS production, which is coincident with the epithelial barrier dysfunction determined by the TEER. A key role played by ROS in the TEER decrease is suggested by the finding that the antioxidant NAC prevents epithelial permeability dysregulation and thus, the *A*. *simplex*-induced oxidative stress would be responsible for the observed alteration of integrity and permeability of Caco-2 cell monolayers. The results align with the observed effect of oxidants on paracellular permeability in Caco-2 cells [43, 44]. The passage of molecules via the paracellular pathway is regulated by phosphorylation/dephosphorylation of proteins in the cytoskeleton and intercellular TJ, and it is thought that oxidative stress dysregulates this enzymatic modulation and finally results in barrier disruption [45, 46].

In this study, occludin and ZO-1 were analysed in Caco-2 cell monolayer exposed to *A*. *simplex* CE to assess whether decreases in TEER could be explained by changes in TJ protein localization. The study results show a remarkable delocalization of occludin and ZO-1 coincident with the maximum decrease in TEER and a relative normalization in TJ localization was observed after 24 hours culture as it had also been observed in TEER measurements. TJ complex is composed of transmembrane proteins, including occludin, junctional adhesion molecules, and members of the claudin family. Occludin is a redox-sensitive protein that provides structural integrity to the TJ since it is an integral component in the barrier function of TJ and interacts with the intracellular protein complex, such as ZO-1, that attaches to the structural elements of the cytoskeleton [46, 47]. Phosphorylation is one of the key players in the interaction of occludin with ZO-1 and other TJ junction proteins. The increase in ROS production can change the phosphorylation pattern of occludin and, as a result, its distribution and interaction with ZO-1 and other TJ proteins would be disturbed, and finally, the epithelial integrity would be comprised [48, 49].

In conclusion, the study results demonstrate that *A*. *simplex* CE induces an early and reversible alteration of integrity and permeability of Caco-2 cell monolayer and that an underlying mechanism of this effect would involve the oxidative stress and disruption of epithelial TJ. In addition, we have shown that Ani s 4 allergen is able to cross the epithelial barrier. These findings could explain the increased intestinal permeability observed in *Anisakis*-sensitized patients and the changes over time in IgE sensitization to *A*. *simplex* allergens. Further, these results could be related to the occurrence of symptoms when sensitized subjects are exposed to fishery products with no live parasite.

## Supporting information

S1 Fig Effect of polymyxin B on the changes in the transepithelial electrical resistance induced by *A*. *simplex* crude extract. Caco-2 cell monolayers were exposed to 2 and 1mg/mL CE in the presence of 30μg/mL polymyxin B. TEER was measured at different time points and the results are expressed as mean ± SD
(TIF)

S2 Fig Effect of the temperature and protease inhibition on the *A*. *simplex*-induced TEER decrease. Caco-2 cell monolayer was exposed to 2mg/mL CE (control) and 2mg/mL CE previously boiled for 30 minutes (Thermostability) or cultured with 2mg/mL CE in the presence of protease inhibitors (Protease inhibition). TEER at 1 and 4 hours of incubation are shown. The results are expressed as median ± SD
(TIF)

S3 Fig Oxygen consumption profiles for Caco-2 2 cells exposed to *A*. *simplex* crude extract. Arrows indicate sequential additions of oligomycin (1 μM), two sequential pulses of FCCP (2 μM and 3 μM) and rotenone and antimycin A (1 μM). The results are expressed as mean ± SD
(TIF)

S4 Fig Extracellular acidification rate profiles of Caco-2 cells exposed to *A*. *simplex* crude extract. Arrows indicate sequential additions of oligomycin (1 μM), two sequential pulses of FCCP (2 μM and 3 μM) and rotenone and antimycin A (1 μM). The results are expressed as mean ± SD
(TIF)
