# Supplementary figures and images for: Anisakis simplex products impair intestinal epithelial barrier function and occludin and zonula occludens-1 localisation in differentiated Caco-2 cells
Source: PLoS Negl Trop Dis. 2020 Jul 6;14(7):e0008462. doi: 10.1371/journal.pntd.0008462 (PMC7365482; doi:10.1371/journal.pntd.0008462)

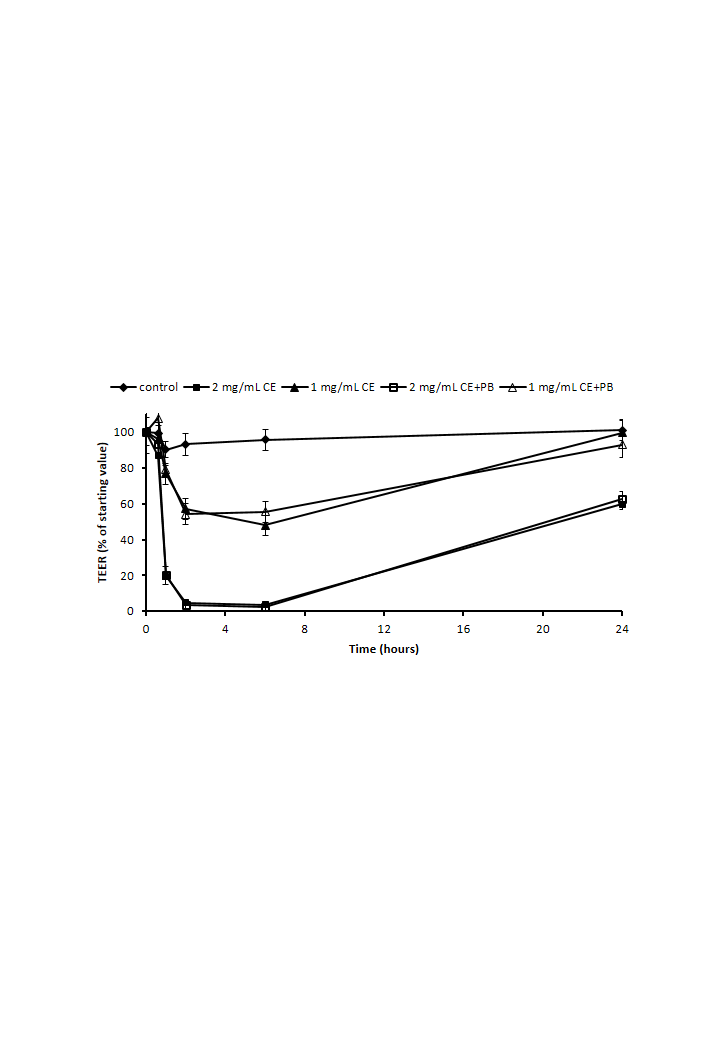

Supplement: S1 Fig — Caco-2 cell monolayers were exposed to 2 and 1mg/mL CE in the presence of 30μg/mL polymyxin B. TEER was measured at different time points and the results are expressed as mean ± SD (TIF) [file pntd.0008462.s001.tif]

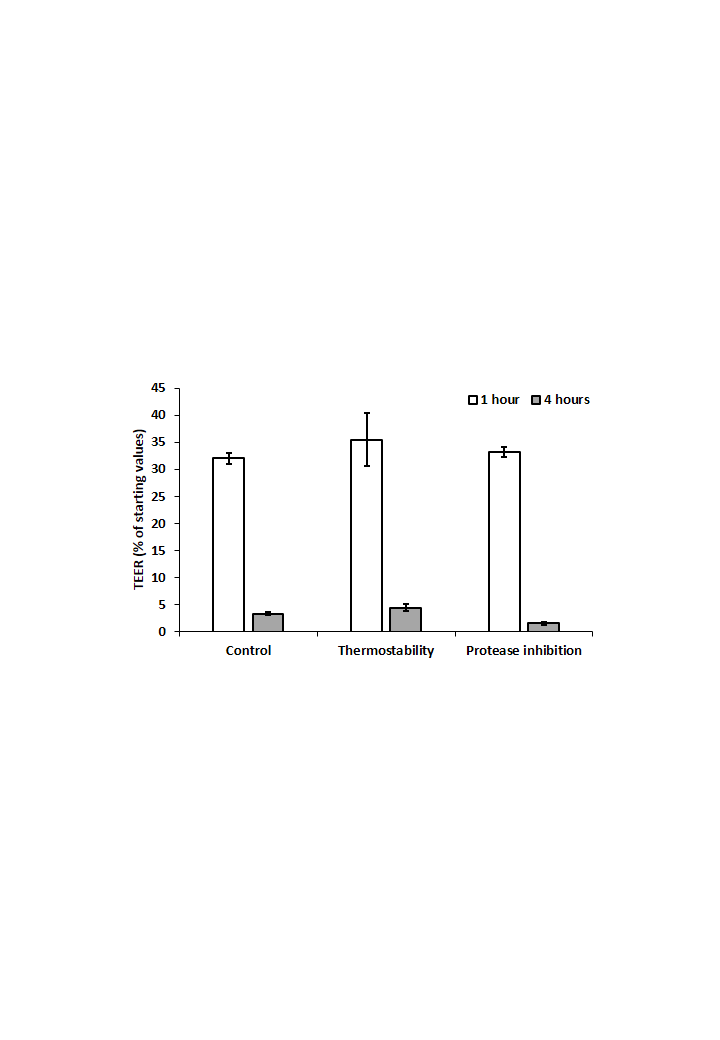

Supplement: S2 Fig — Caco-2 cell monolayer was exposed to 2mg/mL CE (control) and 2mg/mL CE previously boiled for 30 minutes (Thermostability) or cultured with 2mg/mL CE in the presence of protease inhibitors (Protease inhibition). TEER at 1 and 4 hours of incubation are shown. The results are expressed as median ± SD (TIF) [file pntd.0008462.s002.tif]

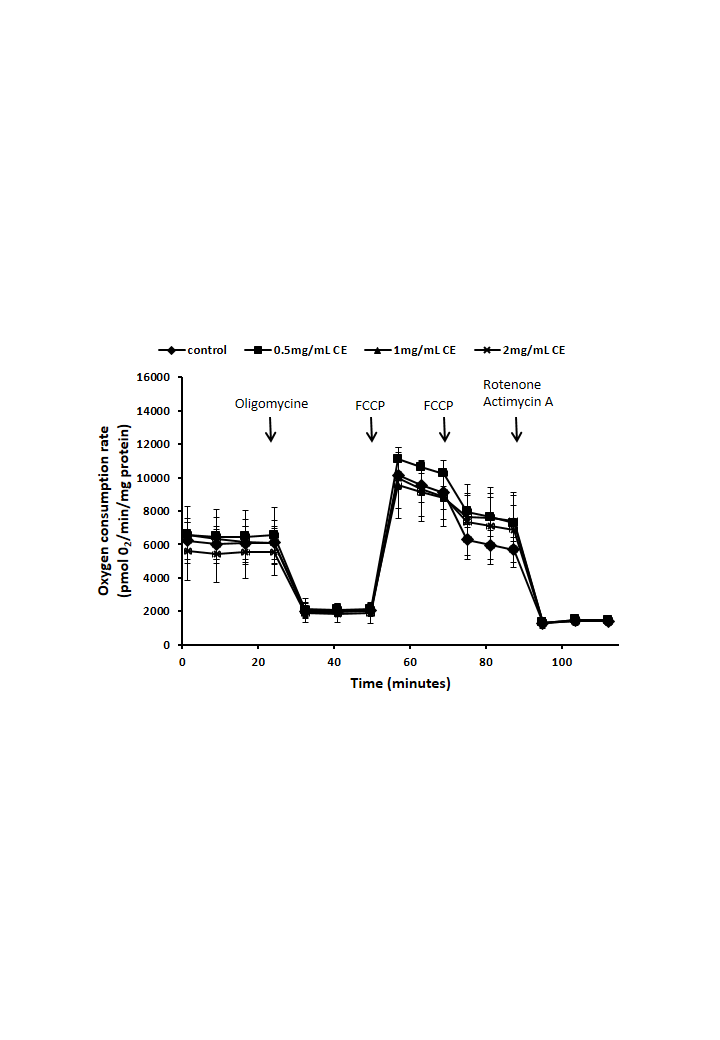

Supplement: S3 Fig — Arrows indicate sequential additions of oligomycin (1 μM), two sequential pulses of FCCP (2 μM and 3 μM) and rotenone and antimycin A (1 μM). The results are expressed as mean ± SD (TIF) [file pntd.0008462.s003.tif]

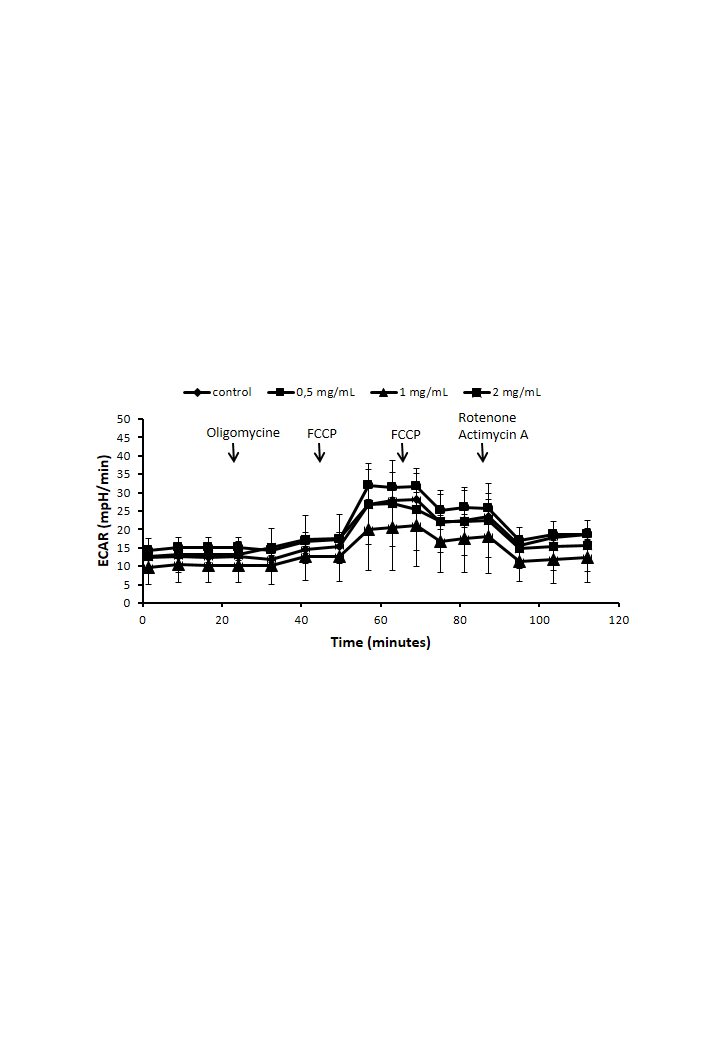

Supplement: S4 Fig — Arrows indicate sequential additions of oligomycin (1 μM), two sequential pulses of FCCP (2 μM and 3 μM) and rotenone and antimycin A (1 μM). The results are expressed as mean ± SD (TIF) [file pntd.0008462.s004.tif]
